# Examining the Influence of Social Interactions and Community Resources on Caregivers’ Burden in Stroke Settings: A Prospective Cohort Study

**DOI:** 10.3390/ijerph182312310

**Published:** 2021-11-23

**Authors:** Yen Sin Koh, Gerald Choon-Huat Koh, David Bruce Matchar, Song-Iee Hong, Bee Choo Tai

**Affiliations:** 1 Research Division, Institute of Mental Health, Singapore 539747, Singapore; Yen_Sin_KOH@imh.com.sg; 2 Saw Swee Hock School of Public Health, National University of Singapore, Singapore 117549, Singapore; ephtbc@nus.edu.sg; 3 Health Services and Systems Research, Duke-NUS Medical School, Singapore 169857, Singapore; david.matchar@duke-nus.edu.sg; 4 Department of Social Welfare, Dongguk University-Seoul, 30 Phildong-ro 1 gil, Jung-gu, Seoul 04620, Korea; songiee25@dongguk.edu

**Keywords:** stroke survivors, caregiver burden, post stroke, care management strategies, assistance to caregivers

## Abstract

Since the introduction of the integrated care model, understanding how social interactions and community resources can alleviate caregivers’ burden is vital to minimizing negative patients’ outcomes. This study (*n* = 214) examined the associations between these factors and caregivers’ burden in stroke settings. It used 3-month and 1-year post-stroke data collected from five tertiary hospitals. Subjective and objective caregivers’ burdens were measured using Zarit burden interview and Oberst caregiving burden scale respectively. The independent variables examined were quality of care relationship, care management strategies for managing patients’ behaviour, family caregiving conflict, formal service usage and assistance to the caregiver. Significant associations were determined using mixed effect modified Poisson regressions. For both types of burden, the scores were slightly higher at 3 months as compared to 1 year. Poorer care-relationship (relative risk: 0.81, 95% confidence interval (CI): 0.70–0.94) and adopting positive care management strategies (relative risk: 1.05, 95% CI: 1.02–1.07) were independently associated with a high subjective burden. Providing assistance to caregivers (relative risk: 2.45, 95% CI: 1.72–3.29) and adopting positive care management strategies (relative risk: 1.03, 95% CI: 1.02–1.04) were independently associated with a high objective burden. Adopting positive care management strategies at 3 months had a significant indirect effect (standardised β: 0.11, 95% CI: 0.01 to 0.20) on high objective burden at one year. Healthcare providers should be aware that excessive care management strategies and assistance from family members may add to caregivers’ burden.

## 1. Introduction

Stroke is the world’s second leading cause of death, and its incidence has risen over the years [1]. According to a global study conducted in 2019, the number of incident stroke cases worldwide was 12.2 million, with 6.55 million deaths related to stroke [1]. Between 1990 and 2019, the number of incident stroke cases increased by approximately 70% worldwide [1]. In Asia, the incidence of stroke is around 116 to 483 per 100,000 individuals annually [2]. Particularly in Singapore, the incident of stroke cases increased from 5890 to 8849 between 2010 and 2019 [3].

Stroke can also result in disability. Approximately 40–45% of stroke survivors experience disability in the first month of stroke [4,5]. Depending on the severity of functional limitation, stroke survivors may require a caregiver to assist in their daily needs. Over the years, there has been an emphasis on the patient–caregiver dyadic relationship in the literature [6]. This trend is due to the increasing evidence that caregivers’ well-being can influence patients’ outcomes [7,8]. For instance, caregivers with poor mental health were more likely to result in the eventual institutionalization of their patients [9].

A concept that is often associated with the patient–caregiver dyadic relationship is caregivers’ burden. It is defined as how caregivers respond physically and emotionally to the changes and demands in the caregiving process [10]. Studies have shown that 25–54% of the caregivers experienced burden during the first six months after stroke [5,11,12,13]. As stroke can occur unexpectedly, caregivers are often unfamiliar with the skills needed to care for the patient [5,14]. Moreover, they may have difficulty coping with the additional responsibilities [5] as well as changes to patients’ physical, cognitive and psychosocial functions [4]. Consequently, they may have a higher risk of developing health problems, such as depression and anxiety [4,15].

Caregivers’ burden can be divided into two aspects: subjective and objective burden. Subjective burden is defined as the emotional and psychosocial consequences of caregiving [14,16]. Objective burden includes the physical caregiving tasks and the time spent in caregiving. These two concepts were often not delineated in existing stroke-related literature [17]. As a result, the combined score generated led to ambiguous findings that were difficult to translate into scientific practice [17].

Many studies have examined the association between caregivers’ burden with patients’ and caregivers’ characteristics [18,19,20]. A systematic review revealed that (i) caregivers’ mental health and (ii) time and effort of caregiving responsibilities were associated with caregivers’ burden [4]. Another meta-analysis showed that patient-related factors with large effect sizes were activity of daily living and anxiety. Furthermore, caregiver-related factors with large effect sizes were depression, anxiety and sense of coherence [10]. However, there are limited studies that explore beyond the patient–caregiver dyadic relationship. Such exploration is pertinent as caregiving is multidimensional and includes, for instance, community support.

The community can support caregivers in their responsibilities through the following approaches: teaching skills to cope with responsibilities, maintaining a good relationship with the patient and getting assistance from family members as well as formal support services [21]. Studies have also utilised concepts such as social capital to explain the networks that caregivers can tap on for support [21]. With the recent advocate for community care [22,23], it becomes relevant to understand whether community support may alleviate caregivers’ responsibilities. In this study, we examined community support in the form of social interactions and community resources. Caregivers’ interpersonal relations with the community and the patient are referred to as social interactions. Community resources refer to tools that caregivers can use to care for patients in their community. These resources can be intangible, such as caregiving skills (e.g., arranging a patient’s surroundings to ensure that it is safe for the patient) taught to caregivers.

Investigating community support for caregivers is applicable to Singapore. Singapore is a country that situates in South-East Asia. It has a multi-ethnic population, consisting of Chinese (75.9%), Malays (15.0%), Indians (7.5%) and other ethnicities (1.6%) [24]. The collectivistic culture in Singapore suggests that the community and social interactions may shape the caregiving experience [25]. Moreover, Singapore is shifting its healthcare model towards an integrated care approach [22]. Hence, it implies the need to understand whether existing social networks and community resources can relieve caregivers’ responsibilities.

Thus, this study aims to examine (i) the trend of caregivers’ burden and utilisation of community resources at three-month and one-year post-stroke, (ii) whether community factors such as social interactions and community resources are associated with measures of caregivers’ burden and (iii) the longitudinal effects of significant determinants on caregivers’ burden. The study divided caregivers’ burden into subjective and objective burden.

## 2. Materials and Methods

### 2.1. Participants and Data Collection

This study utilised data from the Singapore Stroke Study (S3), a longitudinal cohort study that recruited participants from December 2010 to September 2013 [26]. The study was approved by SingHealth Centralized Institutional Review Board (Ref.: 2010/724/A) and the National Health Group Domain Specific Review Board (Ref.: A/10/690). The participants were recruited from five tertiary hospitals: Changi General Hospital, Khoo Teck Puat Hospital, Tan Tock Seng Hospital, Singapore General Hospital and National University Hospital. Participants were recruited if they were (i) Singaporeans or permanent residents; (ii) aged 40 years and above; (iii) staying in Singapore for the following year; (iv) recently diagnosed with stroke (having stroke symptoms within four weeks before admission) by a doctor and/or confirmed by CT/MRI scan of the brain as well as (v) not globally aphasic [26]. All patients who met the inclusion criteria were approached for the study.

Data collected in S3 were obtained from the participants and, if applicable, caregivers at five time points: baseline (at hospital admission), three-month, six-month, nine-month and one-year post-stroke. The data analysed in this study were based on three-month and one-year post-stroke, as the relevant variables were collected at these time points. Data at baseline, three-month and one-year post-stroke were collected via face-to-face interviews with paper-and-pencil. Written consent was obtained from both patients and caregivers after the study procedure was explained.

Only patients with a caregiver were included in this study. A caregiver could either be a family member or friend, aged more than 21 years taking care of the patient’s needs without full financial payment [26]. The following patients were excluded: (i) wrong diagnosis of stroke; (ii) patients who either changed caregiver or had no caregiver at three-month follow up; (iii) lost to follow up at three months and (iv) no information on a change in caregiver at three-month. For patients who changed caregiver at one year, caregiver’s data at three-month post-stroke was included but not at one-year.

### 2.2. Dependent Variables

The outcome measure was caregivers’ burden, which was divided into subjective and objective caregiver burden. Subjective burden was measured using Zarit burden interview (ZBI), a self-reported questionnaire reflecting caregivers’ perspectives about negatively phrased statements on caregiving. With a previous local study showing good validity and reliability (Cronbach’s alpha = 0.93), the shorter 12-item version was used in S3. The responses were recorded based on a 5-point Likert scale: 0 = Never, 1 = Rarely, 2 = Sometimes, 3 = Quite frequently and 4 = Nearly always [27]. The total score for the scale ranges from 0 to 48, with a higher score representing a higher caregiver burden [28].

Objective burden was measured using the Oberst caregiving burden scale (OCBS), a 15-item self-reported questionnaire that examines the time spent and difficulty faced by caregivers with specific tasks. The total score ranges from 15 to 75 [29]. In S3, only the time aspect of the measure was assessed. It was shown previously to have good psychometric properties with stroke survivors [29]. The responses were recorded based on a 5-point Likert scale ranging from 1 = None to 5 = A great amount [29]. A higher score indicates that the caregiver spent a longer time performing the tasks [29].

### 2.3. Independent Variables of Interest

For social interactions, the following determinants at three-month and one-year post-stroke were examined: (i) quality of care relationship between the patient and the caregiver as well as (ii) family caregiving conflict. In the case of community resources, the following factors at three-month and one-year post-stroke were studied: (i) adopting care management strategies, (ii) use of formal services and (iii) assistance provided to the caregiver.

Quality of care relationship between the patient and the caregiver was captured using a 4-item questionnaire from the University of Southern California Longitudinal Study of Three-Generation Families [30]. It measures closeness in a relationship, communication, similarity in life perspective and the extent to which patient and caregiver get along [30]. The responses were captured using a 5-point Likert scale: 1 = Not at all, 2 = Somewhat, 3 = Fairly and 4 = Very. Responses for the third question (similarity in life perspective) were not captured in this study, as local respondents had difficulty understanding the question during the pilot testing phase. After excluding the third question, the range of the composite score was between 3 to 12. A higher score represents a better quality of care relationship [30].The internal consistencies in this dataset were excellent. The Cronbach’s alpha at three-month and one-year post-stroke were 0.88 and 0.95 respectively.

Family caregiving conflict was captured with an 8-item scale examining the attitudes and actions towards both the patient and the caregiver [31]. The responses were recorded with a 4-point Likert scale with 1 = Strongly agree and 4 = Strongly disagree [31]. The range of score is between 4 to 16 for both patients’ and caregivers’ aspects, with a higher score showing more family conflicts [31]. The internal consistencies of the sub-scales in this dataset were excellent, with Cronbach’s alpha between 0.98 to 0.99.

Care management strategies refer to how caregivers handle patients’ behaviour [32]. It was measured using the 20-item revised dementia management strategies instrument. It consists of twelve positively phrased and eight negatively phrased questions, with the responses recorded using a 5-point Likert scale: 1 = Never, 2 = Seldom, 3 = Sometimes, 4 = Often and 5 = Most of the time [33]. The composite score ranges from 12 to 60 for the positive dimension and 8 to 40 for the negative dimension [33]. A higher score represents greater adoption of care management strategies. Both dimensions have good reliability and validity for local caregivers in the dementia setting [33]. Though the questionnaire has not been utilised in stroke settings, the internal consistencies in this dataset were excellent, with Cronbach’s alpha between 0.85 to 0.95.

Usage of formal services was recorded as “Yes” if the caregiver utilised either one of the following services: adult day care centre, caregiver support services, community case management services/counselling services/family service centres, day rehabilitation centre, home help services, home medical/nursing services, home therapy services, neighbourhood links/senior activity centres and respite care at community hospital/nursing home [9].

Assistance provided to caregiver was recorded as “Yes” if there was assistance for either one of the following caregiving tasks in the past three months: assisting in financial matters, bathing, doing minor healthcare activities, dressing, eating, getting in or out of bed or chair, performing light housework, preparing meals, shopping, taking medicine, using the bathroom, using telephone, using transport and walking. Anyone, including family members and domestic workers, could provide assistance to the caregiver.

### 2.4. Control Variables

From the literature, established caregiver-related factors related to caregivers’ burden were anxiety, sense of coherence, emotional distress, amount of time provided to caregiving and number of caregiving tasks performed [4,10]. For established patient-related factors, they were activities of daily living (ADL) and anxiety [4,10]. Caregivers’ depression and patients’ ADL were the control variables that were available in this dataset.

Patients’ demographics collected in the study included age, sex and ethnicity (Chinese, Malay, Indian, other). Age, sex, ethnicity (Chinese, Malay, Indian, Other), educational qualification (no qualification, primary, secondary, post-secondary/polytechnic, university), employment status (working full-time, working part-time, homemaker/housewife, unemployed and retired), and relationship with the patient (husband, wife, brother, sister, son, daughter, grandson, granddaughter, brother-in-law, sister-in-law, son-in-law, daughter-in-law, cousin, friend, neighbour and other) were among the demographics collected for caregivers in the study. For employment status, ‘homemaker/housewife’, ‘unemployed’ and ‘retired’ were all categorized as unemployed. In terms of caregivers’ relationship with patients, caregivers who were the patient’s husband or wife were classified as spouses. Caregivers who were the patient’s son or daughter were categorized as children. Caregivers who had other relationships with the patients were classified as others because the sample size was too small for further stratification (*n* = 20). As patients’ and caregivers’ demographics were not established factors of caregivers’ burden [4], only demographics that were significant in the bivariate analysis were considered as control variables.

Patients’ ADL was measured by Shah-modified Barthel index (BI), a 10-item instrument assessing the following activities: personal hygiene, bathing, dressing, feeding, toileting, bowel control, bladder control, transfers, ambulation or wheelchair dependent and climbing stairs [34]. The responses for each item were 1 to 5, with a higher score representing less dependence [34].

Caregivers’ depression was determined using 11-item Center for epidemiologic studies depression (CES-D) scale [35]. The responses were recorded using 3-point Likert scale: 1 = None/Rarely, 2 = Sometimes and 3 = Often. The total score ranges from 11 to 33, with a higher score representing more depressive symptoms [35].

### 2.5. Statistical Analysis

For descriptive statistics, patients’ and caregivers’ characteristics were reported at baseline. The time-varying variables were reported at two time points: month three and year one. Mean (standard deviation) or median (interquartile range) was presented for continuous variables depending on the data distribution. Counts and percentages were reported for categorical variables. Bivariate analyses of demographic variables with the respective outcomes were performed via the unadjusted mixed effect modified Poisson regression model by regarding each demographic variable individually in the model.

Before performing regression modelling, ZBI score was dichotomized into low and high burden at an established cut-off score of 17 [27]. For OCBS, as there was no established cut-off score, the score was dichotomized using a data-driven approach. Low and high objective burdens were determined at the cut-off score of 30, which was approximately the median score at both time points.

Mixed effect modified Poisson regression with random intercept was used to identify determinants for ZBI and OCBS, to consider the possible intra-individual correlation in outcomes for repeated measurements at three-months and one-year post-stroke. The inclusion of random intercept allowed the separate estimation of intercepts for each individual. For both outcomes, bivariate analyses were conducted to identify significant variables for the multivariable models. Variable selection of the multivariable models was conducted in a stepwise manner using the Wald test where only variables that were significantly associated with the outcome were retained based on the principle of parsimony, in addition to the control variables that were specified a priori for inclusion in the multivariable model. Thus, the final model for each outcome included all the significant variables of interest, adjusted for established confounders (caregivers’ CES-D and patients’ Barthel index) and the effect of time.

To examine the causal relationship between significant determinants at three-month and one-year outcomes, pathway analyses were conducted using structural equation modelling as a basis. As the assumption of multivariate normality was violated, the asymptotically distribution-free (ADF) method was implemented for the analyses [36]. The models were evaluated using the following metrics: (i) comparative fit index (CFI) > 0.90, (ii) root mean squared error of approximation (RMSEA) < 0.05 for a good model and <0.08 for a close fit model and (iii) standardized root mean squared residual (SRMR) < 0.08 [36]. Determinants of ZBI and OCBS, which were significant in the bivariate analysis, were further considered for inclusion in the path analysis. Further refinements were made to path analysis to identify the most appropriate model based on the model fit statistics. Standardised estimates, *p*-values and R-square were generated from the final model.

All analyses were based on a two-sided test at a 5% significance level and performed using Stata/IC 16.0 (College Station, TX, USA) and R version 4.0.3. Mixed effect modified Poisson regression was implemented using mepoisson command with robust variance estimator. Complete case analysis was implemented for the analyses, except when one-year data was missing due to loss to follow-up or a change in caregiver.

## 3. Results

**Figure 1:**
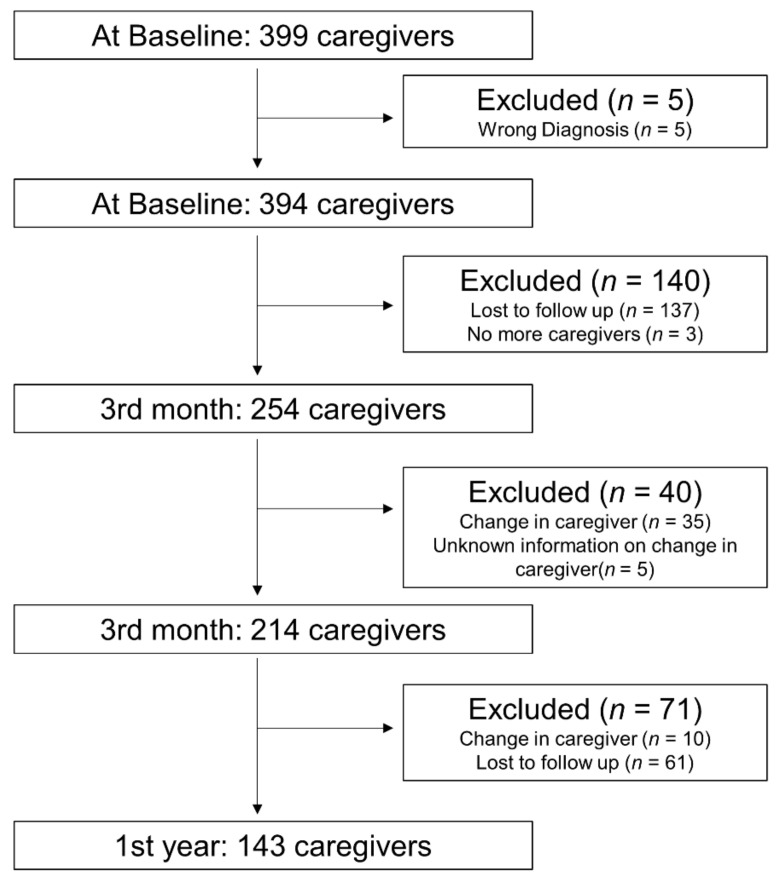
Study flowchart.

Three hundred and ninety-nine caregivers were identified from S3. After excluding ineligible caregivers (Figure 1), 214 caregivers were included in the analysis. At one-year post-stroke, 10 patients changed caregiver and 61 caregivers were lost to follow-up.

The mean age of patients (Table 1) was 63 years (SD: 11.5). Most patients were male (64%), ethnic Chinese (55.6%) and had moderate ADL during admission (median BI (Interquartile range, IQR): 75 (40–94.5)). The mean age of caregivers (Table 1) was 49 years (SD: 13). Most of the caregivers were female (72.4%), ethnic Chinese (53.8%), had secondary school qualifications (41.8%), unemployed (46.7%) and were the spouse (55.9%) of the patient. More than 50% of the caregivers had low subjective burden at baseline (median ZBI (IQR): 11 (6–19)) (Table 1). For caregivers’ depression at baseline, the median CES-D was 7 (IQR: 3–9) (Table 1). Bivariate analyses revealed that patients’ sex (*p*-value = 0.004), patients’ age (*p*-value = 0.038), caregivers’ ethnicity (*p*-value = 0.015) and caregivers’ relationship with the patient (*p*-value = 0.034) were significantly associated with objective burden. Subjective burden was not significantly associated with the variables presented in Table 1.

Table 2 shows the summary statistics for the independent and dependent variables at three-month and one-year post-stroke. The number of caregivers at three-month post-stroke (*n* = 214) was higher than one-year post-stroke (*n* = 143). The ZBI score at three-month post-stroke (median: 7, IQR: 5–13) was slightly higher, as compared to one-year post-stroke (median: 5, IQR: 0–11). Similarly, OCBS at three-month post-stroke (median: 31, IQR: 23–42) was slightly higher, as compared to one-year post-stroke (median: 29, IQR: 19–37).

When the outcome measures were dichotomized (Table 2), a higher proportion of caregivers experienced high subjective burden at one-year (15%) as compared to three-month (12.3%). The proportions of caregivers with high objective burden were similar at three-month (53.6%) and one-year post-stroke (48.9%).

For community resources (Table 2), the mean score for positive care management strategies was higher at three-month (mean: 36.1, SD: 10.8) as compared to one-year (Mean: 31.1, SD: 12.2). The median scores for negative care management strategies were similar at both timepoints (median at three months: 8 (IQR: 8–13), median at one year: 8 (IQR: 8–13)). Most caregivers required assistance for their responsibilities at three-month (65.2%) and one-year (53.9%) post-stroke but did not engage formal service in providing care for the patients (three months: 72.1%, one year: 65%).

### 3.1. Factors Associated with Caregivers’ Burden

Examining the results in unadjusted models, ZBI score was significantly associated with the following determinants: assistance to caregiver, quality of care relationship, positive and negative care management strategies, attitudes and actions towards the patient, caregivers’ CES-D and patients’ Barthel index (Table 3). For OCBS, the unadjusted models showed that the following determinants were significant: assistance to caregiver, positive and negative care management strategies, attitudes and actions towards both the caregivers and the patient, use of formal services, caregivers’ CES-D and patients’ Barthel index (Table 4).

The multivariable model revealed that quality of care relationship, positive care management strategies, caregivers’ CES-D and effect of time were significantly associated with ZBI score (Table 3). Having a better quality of care relationship between the patient and caregiver was associated with low caregivers’ subjective burden (relative risk (RR): 0.81, 95% confidence interval (CI): 0.70 to 0.94). Adopting positive care management strategies (RR: 1.05, 95% CI: 1.02 to 1.07) and depression of caregivers (RR: 1.23, 95% CI: 1.17 to 1.29) were associated with high caregivers’ subjective burden. There was an increased risk of high caregivers’ subjective burden at one-year post-stroke relative to three-month post-stroke (RR: 2.30, 95% CI: 1.40 to 3.78).

For OCBS, assistance to caregiver, positive care management strategies and patients’ Barthel index were significant determinants in the multivariable model (Table 4). Having assistance to caregiver (RR: 2.45, 95% CI: 1.72 to 3.49) and adopting positive care management strategies (RR: 1.03, 95% CI: 1.02 to 1.04) were associated with high caregivers’ objective burden. Better functional status of patient (RR: 0.994, 95% CI: 0.991 to 0.997) was associated with low caregivers’ objective burden.

### 3.2. Pathway Analysis

**Figure 2:**
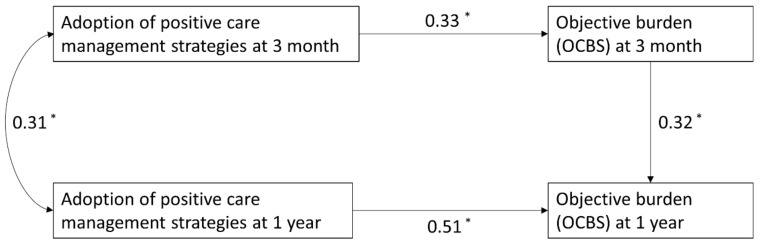
Pathway analysis with OCBS as the outcome. Standardised path coefficients were presented. * *p*-value < 0.05.

The path analysis revealed that the indirect relationship between adoption of positive care management strategies at three months and OCBS at one year was mediated by OCBS at three months (Figure 2). For an increment of one SD in the score for positive care management strategies at three-month post-stroke, OCBS at one-year post-stroke increased by 0.11 points (95% CI: 0.01 to 0.20) through OCBS at three-month post-stroke (Table 5).

In addition, a direct relationship was observed between adopting positive care management strategies at one year and OCBS at one year (Figure 2). An increment of one SD in the score for positive care management strategies at one-year post-stroke was associated with an increment in OCBS at one-year post-stroke of 0.51 points (95% CI: 0.37 to 0.65) (Table 5). This model provided a good fit, as shown from the goodness-of-fit statistics (Table 5) and explained 39.2% of the variation in OCBS at one-year post-stroke.

## 4. Discussion

The shift towards an integrated care model indicates a need to understand whether the community can support caregivers in their responsibilities. Our results showed that the median scores for objective and subjective burden were slightly lower at one-year post-stroke relative to three-month post-stroke. However, the risk of high subjective burden was significantly higher at one-year post-stroke than three-month post-stroke after adjustment for quality of care relationship, adopting positive care management strategies, caregivers’ depression and patients’ functional status. While it is difficult to explain this reversal in trend, it may be due to the different caregiving experiences of caregivers who had a substantial increase in subjective burden over time. However, this possibility could not be explored further due to the small sample size (*n* = 15).

For community resources, while most caregivers had assistance to their role, only a minority utilised formal services at three-month and one-year post-stroke. The finding corroborates with two Asian studies, which mentioned that caregivers preferred assistance from informal supports, such as family members [37,38]. The reasons were lack of trust, financial issues, the limited information available for formal services and communication barriers [37,38]. Our results showed the need to understand the reasons behind the low utilisation. Additionally, services can be improved to meet the caregivers’ needs.

From the regression models, adopting positive care management strategies was significantly associated with both high subjective and objective burdens. Moreover, adopting positive care management strategies at three-month post-stroke could indirectly increase objective burden at one-year post-stroke via objective burden at three-month post-stroke. This indirect effect implies that adopting positive care management strategies during the early stroke recovery period might have a long-term effect on the objective burden in the late stroke recovery period.

Related studies showed divergent findings, presenting no significant effect or significantly lower subjective burden with better care management strategies [17,39,40,41]. However, our results were counter-intuitive and not in parallel with the literature in general. Nonetheless, a stroke-related study had similar findings in which caregivers with higher self-efficacy were more likely to have a higher burden. These caregivers might aim for higher goals, spend more effort, and persist longer relative to those with lower self-efficacy [40]. With the additional time and effort taken for caregiving, it might result in higher objective and subjective burdens.

For subjective burden, a better quality of care relationship was also significantly associated with a low subjective burden. Other studies had also documented that having a better caregiver–patient relationship lowered subjective burden [42,43]. The trend might be due to the concept of reciprocity, which is the exchange of emotional support between the caregiver and patient [43,44]. This exchange motivated the caregivers and improved their sense of competence in their role [43,44]. In Asia, caregivers might also view their role as an expression of love due to values such as filial piety [38]. These positive perceptions that they acquired from their role might lower their subjective burden [45].

A high objective burden was also significantly associated with providing assistance to caregivers. Although the findings seem unexpected, caregivers might experience objective burden when receiving informal support due to Asian social norms, such as filial piety and a sense of obligation [38]. Such social norms made the caregiver unwilling to receive assistance even from other family members and friends [38]. When they received informal supports, they might over-compensate to fulfil patients’ demands. By doing so, they hoped to regain patients’ trust and alleviate their sense of guilt [38]. The findings could also be explained by the fact that the multivariable regression model did not account for caregiving intensity, such as the time required to provide care.

Our findings imply that adopting positive care management strategies can impose high subjective and objective burdens. Assisting the caregivers may also increase objective burden. Hence, healthcare providers should be mindful that implementing excessive care management strategies and having more support for the caregivers beyond what is needed may impose additional caregivers’ burden. As these findings did not corroborate with the literature, future qualitative studies could be conducted to understand these findings. Moreover, subjective and objective burdens may be distinct concepts. Such distinction was implied in the result, as different determinants were observed for both types of burden. However, this finding should be verified by performing analysis, such as CFA (confirmatory factor analysis) on studies with larger sample sizes. Knowing that these burdens are different concepts may help to tailor better interventions for caregivers and stroke survivors [17].

The study has limitations that need to be recognized. One limitation was the inability of the study to examine the positive caregiving element. Studies in other illness settings showed that caregivers could experience positive gain and burden simultaneously [46]. For instance, a study that examined gains among caregivers in dementia settings revealed that adopting positive care management strategies was significantly associated with caregivers’ gain [47]. Coupled with our results, these findings imply that adopting positive care management strategies can be significantly associated with both caregivers’ gain and burden. Hence, both positive and negative aspects of caregiving are pertinent in understanding the caregiving experience.

A second limitation was the inability to include other established risk factors in the analysis, such as caregiving intensity. As the data was obtained from a secondary source, the analysis could not include other established determinants that were not collected. Thirdly, there might be selection bias and limited generalizability. Caregivers were excluded if they were either lost to follow up or did not complete the questionnaires. Fourthly, although the direction of association was assumed based on the stress process model, the associations found from the regression analyses could not support causality [31]. Fifthly, while multiple testing may be a concern when two scales were used as outcomes, subjective and objective burdens are distinct concepts, as evidenced by literature [14,16]. Hence, it is appropriate to analyse these concepts separately. Finally, the small sample size made it difficult to generate complex pathway analyses. Hence, conclusions related to causal relationships were limited in this study.

While this study has certain limitations, it also has several strengths. Firstly, it contributes to the limited number of Asian studies on caregivers’ burden in stroke settings. Moreover, it is one of the few Asian studies that examined community support. Investigating community support is relevant given the collectivistic culture of Asian countries and the shift towards an integrated care model [25]. Secondly, the analysis used modified Poisson regression model to determine relative risk [48]. Estimating relative risk is more intuitive than odd ratios, which can overestimate relative risk especially if the events are common [48].

## 5. Conclusions

Our study showed that poorer quality of care relationship and adopting more positive care management strategies were associated with high subjective burden. Providing more assistance and adopting more positive care management strategies were associated with high objective burden. Our study contributes to scientific knowledge by informing healthcare providers that caregivers’ burden may increase by (i) encouraging excessive adoption of positive care management strategies and (ii) having assistance for the caregiver beyond what is required. Further Asian qualitative studies are needed to examine the interactive mechanism behind these counter-intuitive associations.

## Figures and Tables

**Table 1:** Baseline characteristics of caregivers and patients.

Characteristics of Patients		*n* = 214
Mean age, year (SD)		62.8 (11.5)
Sex	Male	137 (64.0%)
	Female	77 (36.0%)
Ethnicity	Chinese	119 (55.6%)
	Malay	70 (32.7%)
	Indian	21 (9.8%)
	Others	4 (1.9%)
Median Barthel Index during admission		75 (40–94.5)
**Characteristics of Patients**		***n*** **= 214**
Mean Age, years (SD)		49.0 (13.0)
Sex	Male	59 (27.6%)
	Female	155 (72.4%)
Ethnicity	Chinese	114 (53.8%)
	Malay	74 (34.9%)
	Indian	19 (9.0%)
	Others	5 (2.4%)
Educational qualification	No qualification	8 (3.7%)
	Primary	50 (23.4%)
	Secondary	89 (41.8%)
	Post-Secondary/Polytechnic	52 (24.4%)
	University	14 (6.6%)
Employment status	Employed Full time	91 (42.9%)
	Employed part time	22 (10.4%)
	Unemployed	99 (46.7%)
Relationship with patient	Spousal	119 (55.9%)
	Children	74 (34.7%)
	Others	20 (9.4%)
Median ZBI score (IQR)		11 (6–19)
Median CES-D (IQR)		7 (3–9)

Abbreviation: CES-D—Center for epidemiologic studies depression, IQR—Interquartile range, SD—Standard deviation, ZBI—Zarit burden Interview.

**Table 2:** Summary statistics for time-varying risk factors and outcome variables.

		3rd Month ^#^ (*n* = 214)	1st Year ^^^ (*n* = 143)
Variables Related to Caregivers		No. (%)	No. (%)
Median ZBI Score (IQR)		7 (5–13)	5 (0–11)
ZBI Classification	Low burden (ZBI < 17)	185 (87.7%)	119 (85.0%)
	High Burden (ZBI ≥ 17)	26 (12.3%)	21 (15.0%)
Median OCBS (IQR)		31 (23–42)	29 (19–37)
OCBS Classification	Low burden (OCBS < 30)	83 (46.4%)	70 (51.1%)
	High Burden (OCBS ≥ 30)	96 (53.6%)	67 (48.9%)
Median Quality of care relationship (IQR)		12 (10–12)	12 (9–12)
Mean Positive Care management strategies (SD)		36.1 (10.8)	31.3 (12.2)
Median Negative Care management strategies (IQR)		8 (8–13)	8 (8–13)
Median Family Conflict: Attitudes and Actions Toward the Caregiver (IQR)		12 (8–16)	10 (8–12)
Median Family Conflict: Attitudes and Actions Toward the Patient (IQR)		12 (8–16)	8 (8–12)
Median CES-D of caregivers (IQR)		4 (2–7)	3 (1–5)
Assistance to caregiver	No	71 (34.8%)	66 (46.2%)
	Yes	133 (65.2%)	77 (53.9%)
Use of formal services	No	150 (72.1%)	89 (65.0%)
	Yes	58 (27.9%)	48 (35.0%)
**Variables Related to Patients**			
Median Barthel Index (IQR)		99.5 (75.5–100)	99 (80.5–100)

Abbreviation: CES-D—Center for epidemiologic studies depression, IQR—Interquartile range, OCBS—Oberst caregiving burden score, SD—Standard deviation, ZBI—Zarit burden interview. ^#^ Number of missing observations at 3-month: ZBI (*n* = 3), OCBS (*n* = 35), Positive care management strategies (*n* = 5), Attitudes and actions toward the caregiver (*n* = 12), Attitudes and actions toward the patient (*n* = 11), CES-D of caregivers (*n* = 5), Assistance to caregiver (*n* = 10), Use of formal services (*n* = 6), Barthel Index (*n* = 2). ^^^ Number of missing observations at one-year: ZBI (*n* = 3), OCBS (*n* = 6), quality of care relationship (*n* = 4), Positive care management strategies (*n* = 2), Attitudes and actions toward the caregiver (*n* = 1), Attitudes and actions toward the patient (*n* = 1), CES-D of caregivers (*n* = 2), Use of formal services (*n* = 6), Barthel Index (*n* = 11).

**Table 3:** Unadjusted and adjusted models for ZBI using mixed effect modified Poisson regression.

		Unadjusted Model		Adjusted Model ^†^	
Variable Related to Caregivers		RR (95% CI)	*p*-Value	RR (95% CI)	*p*-Value
Assistance to caregiver	No (Reference)				
	Yes	2.65 (1.28–5.51)	0.009 *		
Quality of care relationship		0.79 (0.69–0.90)	<0.001*	0.81 (0.70–0.94)	0.004 *
Positive Care management strategies		1.03 (1.00–1.05)	0.028 *	1.05 (1.02–1.07)	0.002 *
Negative Care management strategies		1.01 (1.00–1.02)	0.015 *		
Attitudes and Actions Toward the Caregiver		0.95 (0.90–1.00)	0.068		
Attitudes and Actions Toward the Patient		0.93 (0.88–0.99)	0.019 *		
Use of formal services	No (Reference)				
	Yes	1.16 (0.67–2.01)	0.600		
CES-D of caregivers		1.21 (1.15–1.28)	<0.001 *	1.23 (1.17–1.29)	<0.001 *
**Variables related to patients**		**RR (95% CI)**	***p*** **-value**	**RR (95% CI)**	***p*** **-value**
Barthel Index		0.987 (0.980–0.993)	<0.001 *	0.994 (0.987–1.001)	0.092
Timepoint	3rd month (Reference)				
	1st year	1.22 (0.75–1.98)	0.421	2.30 (1.40–3.78)	0.001 *

Abbreviation: CES-D—Center for epidemiologic studies depression, CI—Confidence interval, RR—Relative risk, ZBI—Zarit burden interview. **^†^** Number of caregivers included for adjusted model = 208. * *p*-value < 0.05.

**Table 4:** Unadjusted and adjusted models for OCBS using mixed effect modified Poisson regression.

		Unadjusted Model		Adjusted Model ^†,‡^	
Variable Related to Caregivers		RR (95% CI)	*p*-Value	RR (95% CI)	*p*-Value
Assistance to caregiver	No (Reference)				
	Yes	2.94 (2.10–4.10)	<0.001 *	2.45 (1.72–3.49)	<0.001 *
Quality of care relationship		1.00 (0.93–1.07)	0.992		
Positive Care management strategies		1.03 (1.02–1.04)	<0.001 *	1.03 (1.02–1.04)	<0.001 *
Negative Care management strategies		1.006 (1.000–1.012)	0.027 *		
Attitudes and Actions Toward the Caregiver		0.973 (0.949–0.998)	0.038 *		
Attitudes and Actions Toward the Patient		0.974 (0.950–0.998)	0.036 *		
Use of formal services	No (Reference)				
	Yes	1.27 (1.01–1.59)	0.041 *		
CES-D of caregivers		1.05 (1.02–1.08)	<0.001 *	1.02 (0.99–1.05)	0.302
**Variables related to patients**		**RR (95% CI)**	***p*** **-value**	**RR (95% CI)**	***p*** **-value**
Barthel Index		0.990 (0.987–0.992)	<0.001 *	0.994 (0.991–0.997)	<0.001 *
Timepoint	3rd month (Reference)				
	1st year	0.91 (0.75–1.11)	0.364	1.11 (0.89–1.38)	0.368

Abbreviation: CES-D—Center for epidemiologic studies depression, CI—Confidence interval, OCBS—Oberst caregiving burden score, RR—Relative Risk. ^†^ Adjusted for ethnicity of caregiver. Patients’ sex, patients’ age and caregivers’ relationship with patient were not included as they were not significant in the multivariable model. Hence, they were removed when the model was refined. **^‡^** Number of caregivers included for adjusted model = 182. * *p*-value < 0.05.

**Table 5:** Standardised path coefficient and goodness-of-fit statistics for objective burden (OCBS) at one year.

Direct Effect	Standardized Coefficient (95% CI)	*p*-Value
OCBS (3rd month)	0.32 (0.16 to 0.47)	<0.001 *
Adoption of positive care management strategies (one-year)	0.51 (0.37 to 0.65)	<0.001 *
**Indirect Effect**	**Standardized Coefficient (95% CI**)	***p*** **-Value**
Adoption of positive care management strategies (3rd month)	0.11 (0.01 to 0.20)	0.025 *
**Fit Statistics**		Value
Root mean squared error of approximation (RMSEA)		0.077
Comparative fit index (CFI)		0.962
Standardized root mean squared residual (SRMR)		0.035

* *p*-value < 0.05.

## Data Availability

The data presented in this study are available on request from the corresponding author. The data are not publicly available due to institutional policies.
